# Nursing Animosities

**Published:** 1880-08

**Authors:** 


How it warps the judgment, how it eats out the soul, how it kindles into fierce flame the worst passions of our nature, how it magnifies to mountain proportions, the veriest trifle which, in better and nobler moments, would have been passed over with an indifference which would not have given it a second thought. How mean it is too, to mope, and fume, and fret for half a night about a little grievance which, if it had been committed with the utmost deliberation, should have been passed over with a magnanimous forgetfulness. But when it is remembered that most frequently the offence is imaginary, or has arisen from thoughtlessness or inadvertence, the poor fretter becomes the object of our commisseration. Human life is too short, it is too holy a thing to be frittered away in cherishing animosities or in recording wrongs.

